# BiLSTM-LN-SA: A Novel Integrated Model with Self-Attention for Multi-Sensor Fire Detection

**DOI:** 10.3390/s25206451

**Published:** 2025-10-18

**Authors:** Zhaofeng He, Yu Si, Liyuan Yang, Nuo Xu, Xinglong Zhang, Mingming Wang, Xiaoyun Sun

**Affiliations:** 1 School of Electrical and Electronic Engineering, Shijiazhuang Tiedao University, Shijiazhuang 050043, China; hezhaofeng@stdu.edu.cn (Z.H.); 1202309048@student.stdu.edu.cn (N.X.); 1202309052@student.stdu.edu.cn (X.Z.); wangmm@stdu.edu.cn (M.W.); 2 School of Traffic and Transportation, Shijiazhuang Tiedao University, Shijiazhuang 050043, China; 2202306011@student.stdu.edu.cn; 3 School of Mechanical Engineering, Shijiazhuang Tiedao University, Shijiazhuang 050043, China; 2202402008@student.stdu.edu.cn; 4 School of Electronic and Control Engineering, North China Institute of Aerospace Engineering, Langfang 065000, China

**Keywords:** multi-sensor data fusion, fire detection, BiLSTM, self-attention, layer normalization

## Abstract

Multi-sensor fire detection technology has been widely adopted in practical applications; however, existing methods still suffer from high false alarm rates and inadequate adaptability in complex environments due to their limited capacity to capture deep time-series dependencies in sensor data. To enhance robustness and accuracy, this paper proposes a novel model named BiLSTM-LN-SA, which integrates a Bidirectional Long Short-Term Memory (BiLSTM) network with Layer Normalization (LN) and a Self-Attention (SA) mechanism. The BiLSTM module extracts intricate time-series features and long-term dependencies. The incorporation of Layer Normalization mitigates feature distribution shifts across different environments, thereby improving the model’s adaptability to cross-scenario data and its generalization capability. Simultaneously, the Self-Attention mechanism dynamically recalibrates the importance of features at different time steps, adaptively enhancing fire-critical information and enabling deeper, process-aware feature fusion. Extensive evaluation on a real-world dataset demonstrates the superiority of the BiLSTM-LN-SA model, which achieves a test accuracy of 98.38%, an F1-score of 0.98, and an AUC of 0.99, significantly outperforming existing methods including EIF-LSTM, rTPNN, and MLP. Notably, the model also maintains low false positive and false negative rates of 1.50% and 1.85%, respectively. Ablation studies further elucidate the complementary roles of each component: the self-attention mechanism is pivotal for dynamic feature weighting, while layer normalization is key to stabilizing the learning process. This validated design confirms the model’s strong generalization capability and practical reliability across varied environmental scenarios.

## 1. Introduction

As one of the primary disasters threatening human life and property, accurate fire detection remains a critical challenge in the field of public safety. The core objective of fire detection technology is to simultaneously achieve a low False Positive Rate (FPR) and a low False Negative Rate (FNR). The FPR refers to the probability of a detector being erroneously triggered in non-fire scenarios, while the FNR denotes the probability of a detector failing to respond during an actual fire event. Enhancing the accuracy and reliability of fire detection methods is of paramount importance for both theoretical research and practical applications [1].

In recent years, with the acceleration of urbanization and the increasing complexity of industrial environments, traditional fire detection methods relying on single-sensor threshold judgments have exposed significant limitations. On one hand, single-dimensional environmental indicators, such as smoke, temperature, or gas concentration, are susceptible to transient noise interference, resulting in high false positive rates (FPR). For instance, water vapor, dust, or even insects can trigger false alarms in smoke detectors [1,2,3]. On the other hand, fire is a dynamically evolving process that often exhibits subtle early-stage characteristics with time-series patterns across multimodal signals. Conventional methods, which typically ignore time-series dependencies, struggle to capture such latent risks, leading to delayed detection or high False Negative Rates (FNR) [4]. Multi-sensor information fusion, which integrates multidimensional environmental indicators such as temperature, smoke, and CO/CO_2_ concentrations, effectively mitigates the perceptual limitations of single sensors [5]. As multi-sensor fusion has become mainstream, various fusion algorithms have been developed to better leverage complementary sensor information.

Solórzano et al. [6] analyzed the performance of gas sensor arrays and proposed a calibration model with long-term predictive capabilities, effectively enhancing the detection accuracy and stability of gas sensor arrays. Li et al. [7] developed a fire detection method for early fire identification, utilizing the normalized concentration ratio of carbon monoxide to carbon dioxide as a key alarm parameter. Chen Shin-Juh et al. [8] proposed a threshold-based fire detection method by calculating the growth rates of carbon monoxide, carbon dioxide, and smoke concentrations. Baek et al. [9] investigated the performance of multi-sensor systems during the fire perception phase, introducing a sensor data processing method based on a similarity matching algorithm. Liu et al. [10] improved weighted information fusion and Kalman filter fusion algorithms, enhancing system reliability by dynamically adjusting sensor weights. Liu et al. [11] proposed a multi-sensor fusion localization method based on Batch Inverse Covariance Intersection (BICI) to address localization drift issues in confined spaces. Wang et al. [12] introduced a weighted fusion algorithm based on an improved Analytic Hierarchy Process (AHP), enabling rapid detection of aircraft fires and improving timeliness and accuracy in complex scenarios such as aviation fire detection. Chen Jing et al. [13] leveraged the graphical knowledge representation and uncertainty handling capabilities of Bayesian Networks (BN) for fire alarm system analysis. Ran et al. [14] proposed a probabilistic statistical framework for multi-sensor data fusion, extending the lifespan of wireless sensor networks while improving data accuracy. Jana et al. [15] developed a novel algorithm that integrates logistic regression, SVM, decision trees, and naïve Bayes classifiers through feature engineering preprocessing, enhancing prediction accuracy and robustness. Baek et al. [16] introduced a new fire monitoring framework employing Support Vector Machines with a Dynamic Time Warping Kernel (SVM-DTWK), which accounts for the time series dynamics of various fire sensor signals. These methods typically rely on precise system models or prior knowledge, exhibiting limited capabilities in handling complex nonlinear relationships.

The Dempster-Shafer (D-S) evidence theory is a powerful tool for handling uncertainty, with its primary advantage being the ability to flexibly fuse information from different evidence sources (sensors) without requiring prior probability information. Ding et al. [17] developed a multi-sensor building fire alarm system, utilizing D-S evidence theory to integrate data from light, smoke, and temperature sensors. Li et al. proposed several improvements, such as optimizing the Basic Probability Assignment (BPA) function or modifying fusion rules, to enhance the reliability of fusion results [18,19]. In tunnel fire detection, Wang et al. introduced a two-level fusion framework that optimizes BPA and employs trust coefficients to address evidence conflicts, significantly improving the accuracy and response speed of fire detection. Su et al. [20] designed a gradient data fusion model for detecting fires in aging communities, reducing misjudgments in traditional systems. Zhang et al. [21] proposed a novel fire detection approach combining Backpropagation (BP) neural networks with D-S evidence theory, further introducing an evidence correction method based on exponential entropy. Despite its strengths in handling uncertainty, the D-S evidence theory can yield counter-intuitive results when fusing highly conflicting evidence, and the appropriate construction of the Basic Probability Assignment (BPA) remains a non-trivial challenge that significantly impacts performance. Moreover, as the classical D-S framework operates on static data snapshots, it lacks the inherent capability to model time-series dynamics. This fundamentally limits its sensitivity to progressive patterns characteristic of early fire development, which are embedded in time-series data.

Artificial intelligence methods represent a current research hotspot and a primary direction for development. Sowah et al. [22] developed a fuzzy logic system using Arduino, integrating data from smoke, temperature, and flame sensors to achieve fire detection. Hong Bao et al. [23] employed a fuzzy inference system to fuse empirical fire signal characteristics and fitted fire data features, deriving the final fire probability. Rachman et al. [24] designed a system capable of expanding the fire detection range, which collects data from multiple sensors and processes it using fuzzy logic methods to enable effective fire detection. Wang Xihuai et al. [25] developed a multi-sensor fire detection algorithm for shipboard fire detection based on a fuzzy neural network. This algorithm employs multi-sensor integration and multi-parameter fusion to achieve multi-level fire alarming for maritime applications. Qu et al. [26] utilized a Backpropagation Neural Network (BPNN) to fuse data from temperature, smoke concentration, and CO concentration, effectively improving the accuracy of fire warnings. Wu et al. [27] enhanced the BPNN by incorporating non-uniform sampling and trend extraction as inputs, improving the model’s ability to distinguish fire signals from environmental interference. Jiang et al. [28] proposed an intelligent fire detection system based on a fuzzy neural network for multi-sensor information fusion. This system integrates data from temperature, smoke, and carbon monoxide sensors—three parameters with distinct fire-related characteristics—and employs an intelligent fuzzy neural network algorithm to determine the probability of a fire occurrence. The advantage of these methods lies in their ability to model complex, non-linear relationships and handle imprecise information. However, fuzzy systems often depend on expert knowledge for rule definition, while neural networks like BPNN can suffer from slow convergence, local minima, and overfitting, and their “black-box” nature hinders interpretability.

Wen et al. [29] analyzed the seasonal adjustment of time-series data for fire alarm reception, summarizing the fundamental characteristics of seasonal factors. This study provides valuable reference for fire departments to optimize the allocation of firefighting resources, thereby enhancing fire prevention and response capabilities. Ryder et al. [30] proposed a hierarchical temporal memory algorithm and discussed its advantages and limitations for fire state determination in continuous learning environments. Yang Li et al. [31] introduced the TCN-AAP-SVM algorithm, which effectively incorporates the time series dimension of sensor data, demonstrating robust classification performance. Nakip et al. [32] proposed a recursive trend prediction neural network (rTPNN) that integrates trend prediction with hierarchical forecasting of sensor data, achieving significant improvements over traditional Support Vector Machines (SVM) and Bayesian networks. Liu et al. [33] developed the EIF-LSTM model, which fuses environmental indicator variations and contextual information, achieving high performance on the NIST dataset. Deng et al. [34] proposed an indoor fire detection method based on multi-sensor fusion and a lightweight Convolutional Neural Network (CNN), resulting in an efficient lightweight CNN for indoor fire detection. Sun et al. [35] employed an Evidence Reasoning (ER) approach combined with a Particle Swarm Optimization (PSO) algorithm to assess dynamic fire risk levels using heterogeneous multi-source data, including fire images, smoke images, temperature, and carbon monoxide concentrations. Zhang et al. [36] introduced a novel anomaly detection method based on dynamic graph neural networks, which fuses time series features and modality-related features extracted from each sensor node into a vector representation. This is further aggregated with spatial features representing the spatial relationships of nodes to identify anomalous states based on the fused features.

Parallel to these advancements in sensor-based methodologies, a distinct technological trajectory has emerged in the domain of visual fire analysis. This alternative approach capitalizes on the proliferation of surveillance infrastructure and leverages computer vision techniques to process and interpret fire-related visual cues. Rather than relying on physicochemical parameters, video-based fire recognition focuses on extracting and analyzing visual features—including flame morphology, chromatic characteristics, and smoke diffusion dynamics—thereby establishing a separate but complementary paradigm for fire identification.

The integration of attention mechanisms has markedly advanced deep learning-based fire detection, especially in complex scenarios. While early approaches relied on handcrafted features—often leading to poor generalization and high false-positive rates—recent research has shifted toward hybrid architectures that combine Convolutional Neural Networks (CNNs) with Vision Transformers (ViTs). These models leverage attention to enhance feature discrimination and contextual modeling. Notably, Kong et al. [37] developed ADE-Net, a dual-encoding network incorporating multi-attention modules, which achieved a Dice coefficient of 90.69% on the FLAME dataset and effectively addressed small-target detection challenges. Wang et al. [38] proposed Fire ViTNet, a lightweight model integrating Mobile ViT and CBAM attention, attaining an F1-score of 87.2% while maintaining computational efficiency. Ullah et al. [39] introduced AEFRN, a hierarchical attention framework that surpassed 99% accuracy on multiple benchmarks. Safarov et al. [40] embedded ViT into YOLOv5s, achieving a Dice coefficient of 90.93% in UAV-based fire and smoke segmentation and demonstrating robustness to occlusions and illumination variations. In infrared image detection, Zhang et al. [41] constructed a dedicated benchmark and proposed a frequency compression method to mitigate spectral bias and leakage, demonstrating the power of frequency-domain processing in enhancing feature discriminability for weak targets.

In video-based fire detection, attention mechanisms have emerged as a pivotal technology for enhancing detection performance, achieving breakthrough improvements through their superior capabilities in feature discrimination and contextual modeling. However, the application of these mechanisms in sensor-based detection systems remains at a preliminary exploration stage, lacking systematic implementation frameworks. Notably, fire sensor data—comprising key parameters such as temperature, TVOC, and CO_2_—inherently possesses time-series and dynamic correlation characteristics, making its structure highly compatible with the sequence-dependency processing logic of attention mechanisms, whose core operations (such as Query-Key-Value dot products and weight allocation) are fundamentally designed to capture dynamic intra-sequence relationships. Therefore, theoretically, developing specialized attention models tailored to the properties of sensor data—such as multi-source asynchrony and time-series dynamics—could significantly enhance detection accuracy and system robustness while maintaining low false alarm rates. This research direction not only holds theoretical significance but also offers clear practical value.

A comprehensive analysis of existing research shows that time-series-trend-based fire detection models (e.g., EIF-LSTM, rTPNN) have made notable advances while still facing persistent limitations in feature fusion. Prevailing architectures lack dynamic time-series attention mechanisms, relying instead on uniform or static fusion strategies that limit adaptive focus on the most discriminative intervals in multivariate sensor streams—particularly the transient yet critical fluctuations in temperature and gas concentrations that characterize incipient fires. Furthermore, existing methods are typically designed for single-scenario deployment and generalize poorly to our multi-scenario dataset, making feature distribution shift a pressing challenge.

To address these issues, we propose BiLSTM-LN-SA, a multi-sensor fire detection framework integrating Bidirectional LSTMs, Layer Normalization, and time-series self-attention. The architecture employs a three-stage design: First, the BiLSTM backbone captures long-range bidirectional dependencies in time-series sensor data. Subsequently, the Layer Normalization module stabilizes feature distributions to mitigate cross-scenario shift and enhance generalization. Finally, a self-attention mechanism dynamically reweights time-series segments to emphasize the most discriminative features. This integrated approach significantly improves detection accuracy and operational robustness in multi-scenario fire monitoring environments.

## 2. The Proposed Method

**Figure 1:**
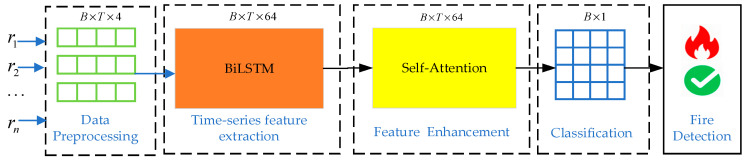
Framework of the BiLSTM-LN-SA model for fire detection.

This section introduces the overall framework of the proposed algorithm, along with the specific structures and implementation details of its components. The framework comprises the following key modules: Data Preprocessing Module, Time-Series Feature Extraction Module, Feature Enhancement Module, and Classification Module. The overall architecture is shown in Figure 1.

### 2.1. Data Preprocessing Module

The data preprocessing Module transforms raw sensor data into a unified, structured dataset through data normalization and time-series sequence construction, enabling subsequent model training and evaluation.

(1) Normalization:

Normalization is applied to sensor data to mitigate the effects of inherent variations in numerical ranges across different sensor types, thereby scaling the values to a common interval and ensuring consistent input for subsequent model training and evaluation. The normalization process is as follows:

(1) $$x_{n}=\frac{r-r_{\min}}{r_{\max}-r_{\min}}$$

where r is the original sensor data value, $r_{max}$ is the maximum value, $r_{min}$ is the minimum value, and $x_{n}$ is the normalized data value, scaled to the range [0, 1]. During normalization, the minimum and maximum values are determined exclusively from the training dataset.

(2) Time-Series Data Generation:

After normalizing, the preprocessed sensor data is used to construct the input matrix X, with dimensions (T,N), where T represents the time steps and N denotes the number of sensor features. In this study, N= 4, corresponding to the features: temperature, TVOC, CO_2_, and NC2.5.

### 2.2. Time-Series Feature Extraction Module

**Figure 2:**
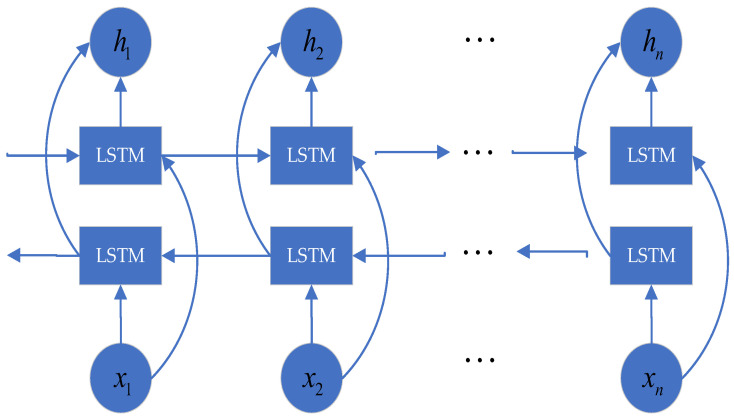
Architecture of the BiLSTM model for fire detection.

The time-series feature extraction module, constructed based on a Bidirectional LSTM (BiLSTM) architecture, serves as a foundational representation layer for multi-sensor data. It is designed to capture dynamic time-series features from input time-series matrices. Unlike traditional Long Short-Term Memory (LSTM) networks, which generate hidden states based solely on historical information, the BiLSTM processes the sequence in both forward and backward directions using two separate LSTM units and integrates the hidden states from both directions. As a result, the model captures contextual information from both past and future time steps, enabling more comprehensive and robust extraction of dynamic time-series features. This capability is particularly essential in fire detection, where sensor data exhibits strong time-series dependencies, and accurate determination of the fire state at any moment relies on integrating information from both preceding and subsequent readings. The architecture of this BiLSTM module is illustrated in Figure 2. The computation process is as follows:

(2) $$\begin{array}{l} \vec{h_{t}}=LSTM(x_{t},{\vec{h}}_{t-1})\\ \overset{\leftarrow }{h_{t}}=LSTM(x_{t},{\overset{\leftarrow }{h}}_{t+1})\\ h_{t}=[\vec{h_{t}};\overset{\leftarrow }{h_{t}}] \end{array}$$

Here, $\vec{h_{t}}$ and $\overset{\leftarrow }{h_{t}}$ denote the hidden states of the forward and backward LSTM at time step t, respectively, each containing D hidden units. The concatenated hidden state at each time step is represented as $h_{t}$. The final output is a time series feature matrix H, formed by concatenating the hidden states across all time steps, with a dimension of 2D.

This resulting matrix, which represents the output of the BiLSTM-based feature extraction module, integrates bidirectional contextual information and hierarchically fused cross-sensor features derived from the simultaneous input of four sensor readings at each time step. It effectively captures long-range dependencies within the multi-sensor sequence, thereby enhancing the recognition of weak yet persistent fire indicators. The architecture allows the model to gradually integrate multi-source information during processing, drawing on complementary environmental signals from all sensors to support its ability to distinguish between false alarms and genuine fire events.

### 2.3. Feature Enhancement Module

**Figure 3:**
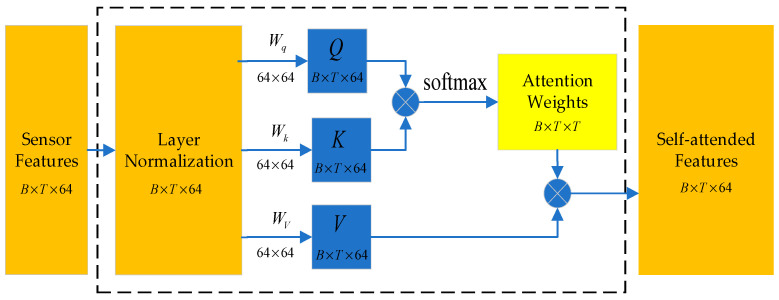
Schematic of the Feature enhancement module for fire detection.

The feature enhancement module incorporates a self-attention mechanism, which enables the model to automatically assign differentiated weights to individual time steps within the input sequence. This design allows the network to selectively focus on segments of the sensor data that are most relevant for fire recognition, as the significance of readings often varies considerably across time. By emphasizing critical intervals in the time series, the self-attention mechanism refines the BiLSTM-output features and extracts key time-step information, thereby contributing to more informed and context-aware decision-making. The architecture of this self-attention mechanism is depicted in Figure 3. The detailed computational procedure is as follows.

First, the time-series features extracted from the sensors are processed by a Layer Normalization module. This operation stabilizes the feature distribution and mitigates internal covariate shift across different environments, ensuring a more stable and efficient learning process for the subsequent attention mechanism. The operations are defined as follows:

(3) $$H_{\mathrm{norm}}=\frac{H-\mu }{\sqrt{\sigma^{2}+\varepsilon }}$$

Here, μ and $\sigma^{2}$ are the mean and variance computed dynamically across the feature dimension of each individual sample within H; This operation does not rely on any statistics accumulated during training, ensuring consistent and stable behavior for both training and inference. ε is a small constant included for numerical stability. The normalization promotes robustness by emphasizing relative anomalies and internal feature relationships rather than absolute feature values, which enhances the model’s adaptability to varying environmental conditions.

These normalized features $H_{\mathrm{norm}}$ are then projected into query (Q), key (K) and value (V) spaces through learnable weight matrices Wq and $W_{k}$, respectively. This projection facilitates the modeling of interactions and correlations across different sensors and time steps. The operations are defined as follows:

(4) $$\begin{array}{l} Q=H_{\mathrm{norm}}W_{q}\\ K=H_{\mathrm{norm}}W_{k}\\ V=H_{\mathrm{norm}}W_{v} \end{array}$$

The attention weight matrix S, which captures the relative importance of each time step, is then computed as the softmax of the scaled dot-products between queries (Q) and keys (K):

(5) $$\begin{array}{l} S\;=\;\mathrm{softmax}\left(\frac{QK^{T}}{\sqrt{d_{k}}}\right)\\ F=S\times V \end{array}$$

The scaling factor $\frac{1}{\surd d_{k}}$ stabilizes the gradients during training. The attention weights S are used to compute a weighted sum of the features V, producing a refined feature representation F that emphasizes task-critical time-series information.

### 2.4. Classification Module

**Figure 4:**
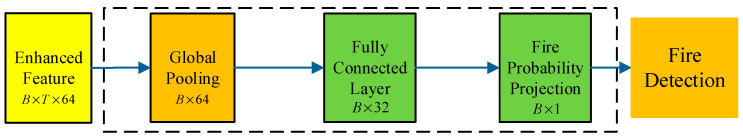
Architecture of the fire probability classification module.

The classification module maps the extracted sensor fused features into a predicted probability of fire occurrence. Its key function is to convert high-level abstract representations into a quantifiable fire risk score. This component consists of a global average pooling layer followed by a classification head network. The overall architecture of this module is illustrated in Figure 4.

(1) Global Average Pooling

This layer aggregates time series features into a unified global representation, preserving the overarching trend of sensor measurements across the entire time window. It summarizes the sequential information by averaging feature values over all time steps, thereby capturing dominant patterns such as sustained temperature increases. The operation is defined as Equation (6):

(6) $$P=\frac{1}{T}\overset{T}{\underset{j=1}{\sum }}F_{\left(:,j\right)}$$

where T is the number of time steps, F is the input feature matrix, and P is the resulting pooled feature vector.

(2) Classification Head

The classification head is designed to map fused features into a discriminative representation space suitable for fire event detection, ultimately producing a probabilistic fire prediction through sigmoid activation.

First, the pooled feature vector P is processed by a fully connected layer with ReLU activation to capture nonlinear patterns inherent in fire-related sensor dynamics. This is formulated as:

(7) $$H_{\mathrm{dense}}=\mathrm{ReLU}(PW_{h}+b_{h})$$

where $W_{h}$ and $b_{h}$ denote learnable weights and biases, and ReLU activation introduces nonlinearity to model complex feature interactions indicative of fire events.

Subsequently, the features are projected onto a fire probability score $\hat{y}\in \left(0,1\right)$ by the Fire Probability Projection Layer (a sigmoid-activated linear transformation):

(8) $$\hat{y}=\sigma (H_{\mathrm{dense}}W_{o}+b_{o})$$

where $W_{o}$ $and\;b_{o}$ are learnable parameters, and σ(·) denotes the sigmoid function. The output $\hat{y}$ represents the estimated probability of a fire event, enabling flexible binary decision-making through application-dependent thresholding.

## 3. Performance Evaluation and Analysis

In this section, we evaluate the performance of our proposed method against several contemporary approaches, namely NP, MLP, rTPNN, and EIF-LSTM, on the task of real-fire detection [32,33].

### 3.1. Experimental Setup and Model Configuration

This section details the dataset, preprocessing procedures, model implementation, and training protocol used to evaluate the proposed BiLSTM-LN-SA model.

#### 3.1.1. Dataset Description

We employ a publicly available fire detection dataset [42] collected using IoT devices, selected specifically for its comprehensive multi-scenario coverage that enables robust evaluation of real-world fire detection capabilities. This dataset’s diverse environmental representation addresses a critical limitation in existing fire detection research, where many studies utilize single-scenario datasets that fail to capture the full spectrum of operational conditions encountered in practical deployment. The dataset encompasses a wide range of environmental conditions, including:

- Normal indoor conditions
- Normal outdoor conditions
- Indoor wood fire within a firefighter training area
- Indoor gas fire within a firefighter training area
- Outdoor wood, coal, and gas grill fires
- High-humidity outdoor environments

This multi-scenario composition is particularly valuable for developing generalized fire detection models, as it exposes the algorithm to diverse sensor response patterns across different combustion characteristics, ventilation conditions, and ambient environmental factors. Unlike approaches trained on single-scenario datasets that may overfit environment-specific artifacts, our method leverages this diversity to learn fundamental fire signatures that transcend specific operational contexts.

The dataset provides synchronized time-series measurements of key environmental parameters, uniformly sampled at 1 Hz with UTC timestamps. The collected variables are: Temperature (°C), Humidity (%), Pressure (hPa), Total Volatile Organic Compounds (TVOC, ppb), equivalent Carbon Dioxide (eCO_2_, ppm), raw sensor readings for Hydrogen and Ethanol, as well as particulate matter mass (PM1.0, PM2.5) and number concentrations (NC0.5, NC1.0, NC2.5). A sample count (CNT) is also provided. A binary Fire Alarm label serves as the ground truth for the classification task. To align with our research objectives and following a careful review of relevant literature [32,33], we focused on four representative features as model inputs: Temperature, TVOC, eCO_2_, and NC2.5.

#### 3.1.2. Time-Series Data Construction

For time-series classification, the data were segmented using a sliding window of 20 time steps with a step size of 2, yielding a 90% overlap between consecutive windows. This configuration maximizes time-series resolution while preserving sufficient sample diversity for effective model training, resulting in a final dataset of 49,888 samples. Each sample represents a 20 s multi-sensor sequence labeled with the fire state at the window’s end.

#### 3.1.3. Model Configuration and Hyperparameters

The architecture and key hyperparameters of the BiLSTM-LN-SA model are specified as follows:

- BiLSTM Module: This module consists of 2 stacked bidirectional LSTM layers. Each LSTM cell in each direction contains 32 hidden units, resulting in a concatenated output dimension of 64.
- Self-Attention Module: We employ a single-head self-attention mechanism. The dimension of the query and key vectors (*d_k_*) is set to 64.
- Classification Head: The fully connected layer following global average pooling comprises 32 neurons with ReLU activation.

#### 3.1.4. Training and Evaluation Procedure

The model training and evaluation were conducted under the following procedure:

Training Setup: The model was trained using the Adam optimizer with an initial learning rate of 0.0001 and Binary Cross-Entropy as the loss function. Training was performed for a maximum of 200 epochs with a batch size of 32, incorporating an early stopping mechanism (patience = 15 epochs) based on validation loss.

Evaluation Method: We employed 10-fold cross-validation on the dataset to comprehensively evaluate the performance of the proposed method. Throughout the training process, the input X takes the form of a three-dimensional tensor with dimensions  B×T×N, where

- B denotes the batch size, set to 32, indicating the number of samples processed in each batch;
- T indicates the number of time steps, which is set to 20, corresponding to the length of consecutive sensor readings per sample;
- N represents the number of sensors (or sensor channels), which is set to 4 in this study.

The output tensor has a shape of B×1, corresponding to the binary fire state prediction for each input sample in the batch.

### 3.2. Results and Analysis

This section presents a comprehensive evaluation and comparative analysis of the proposed BiLSTM-LN-SA model against several baseline methods, including NP, MLP, rTPNN, and EIF-LSTM. To ensure a thorough assessment, multiple performance metrics are employed: classification accuracy, confusion matrix analysis (TPR, FNR, TNR, FPR), F1-score, and ROC curves with AUC values. Additionally, the impact of time-series resolution on detection performance is investigated through a step-size analysis. Ablation studies are conducted to quantify the individual contributions of the Layer Normalization (LN) and Self-Attention (SA) modules. Finally, a holistic discussion synthesizes the complementary roles of LN and SA, elucidating their synergistic effects in enhancing model robustness and discriminative power under multi-scenario conditions.

#### 3.2.1. Metrics of Accuracy

Accuracy, defined as the proportion of correctly classified samples, is a fundamental performance metric for classification tasks. In fire detection, it directly reflects the detection efficacy and generalization capability of an algorithm. The “Mean” values in the tables represent the average accuracy from 10-fold cross-validation on the training and test sets, quantifying the model’s overall performance. The “Std” (standard deviation) indicates the dispersion of results across the folds, where a lower value denotes higher consistency and robustness.

As shown in Table 1, BiLSTM-LN-SA demonstrates advantages in both mean accuracy and standard deviation. Its mean test accuracy reaches 98.38%, outperforming EIF-LSTM (95.30%) by 3.08 percentage points, rTPNN (93.85%) by 4.53 percentage points, and significantly surpassing MLP (88.27%) and NP (80.12%). This improvement is primarily attributed to the model’s enhanced capability to extract and integrate characteristic fire signatures from multi-sensor time-series data. Specifically, our approach effectively identifies distinctive fire-related patterns—including non-linear temperature trajectories, gas concentration changes rates, and multi-sensor correlation dynamics—that reliably distinguish fire events from environmental anomalies. This capability significantly strengthens the discriminative power for accurate fire identification across diverse operational scenarios.

To further validate the advantage of multi-sensor fusion, we compared the performance against systems using individual sensor data, as presented in Table 2. A noticeable performance gap exists, with the highest test accuracy among single-sensor data being only 85.75% (TVOC). This gap arises because univariate physical signals are insufficient to characterize the multi-stage dynamics of fire events. The limitation in feature diversity inherently restricts their discriminative capability.

The accuracy results confirm the effectiveness of the BiLSTM-LN-SA model. The combination of a high mean and a low standard deviation preliminarily validates the advantage of its architecture in integrating multi-sensor time-series information and enhancing model robustness.

#### 3.2.2. Metrics of Confusion Matrix

The confusion matrix serves as a fundamental tool for evaluating classification performance by comparing predicted outcomes against actual instances. In contrast to accuracy, which only considers the overall probability of correct classification, the confusion matrix addresses accuracy’s limitation by providing a detailed breakdown of different types of classification errors. The confusion matrix in this study includes four key metrics: True Positive Rate (TPR), False Negative Rate (FNR), True Negative Rate (TNR), and False Positive Rate (FPR). TPR reflects the rate of actual fires correctly identified, while FNR indicates the rate at which fires are missed. Conversely, TNR measures the correct identification of non-fire events, and FPR represents the rate of false alarms. These four metrics obey the following relations: TPR+FNR = 100% and TNR+FPR = 100%.

The confusion matrix metrics of BiLSTM-LN-SA and other multi-sensor methods are compared in Table 3. The proposed model demonstrates superior performance across all four key metrics: it achieves a TPR of 98.15% and an FNR as low as 1.85%. Compared to EIF-LSTM (FNR = 4.80%), this represents a reduction of 2.95 percentage points in the false negative rate. Additionally, BiLSTM-LN-SA attains a TNR of 98.50% and an FPR of only 1.50%, which is 1.6 percentage points lower than that of EIF-LSTM (FPR = 3.10%). These results indicate that BiLSTM-LN-SA improves fire detection accuracy and reduces the false alarm rate.

To further illustrate the effectiveness of the multi-sensor fusion strategy, the BiLSTM-LN-SA model was compared with various single-sensor methods, as listed in Table 4. The results indicate inherent limitations in single-sensor approaches. For example, the temperature-based approach achieves a TPR of only 28.30%, whereas BiLSTM-LN-SA exceeds it by over 69.85 percentage points. Although certain single-sensor methods achieve a relatively low FPR (e.g., the TVOC-based method has an FPR of 0.90%), their TPR remains considerably low (e.g., 85.40%), making it difficult to balance detection rate and false-alarm rate effectively.

In summary, BiLSTM-LN-SA achieves an effective balance between the missed detection rate and the false alarm rate, thereby offering a reliable solution for intelligent fire detection in complex scenarios.

#### 3.2.3. Metrics of F1-Score

The F1-score is a comprehensive metric used for evaluating classification performance, defined as the harmonic mean of precision and recall. It is calculated as follows:

(9) $$F_{1}=2\cdot \frac{\mathrm{Precision}\cdot \mathrm{Recall}}{\mathrm{Precision}+\mathrm{Recall}}$$

In the context of fire detection, precision indicates the proportion of correctly identified fire events among all alarms triggered, while recall (also known as sensitivity) measures the model’s ability to detect actual fires, thereby directly influencing the risk of missed detections.

The F1-score balances both the accuracy and completeness of positive-class predictions, with values ranging between [0, 1]. Values closer to 1 indicate better overall classification performance.

**Figure 5:**
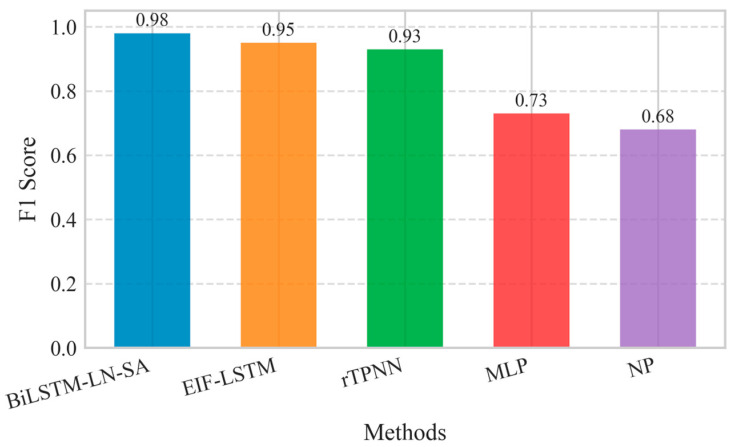
Comparison of F1-scores across different multi-sensor fire detection methods.

As shown in Figure 5, the multi-sensor fusion method BiLSTM-LN-SA achieved an F1-score of 0.98, which is higher than that of other methods. These results demonstrate that BiLSTM-LN-SA achieves a good balance between precision and recall, reflecting its capability for comprehensive classification performance.

The high F1-score underscores the effectiveness of BiLSTM-LN-SA in leveraging cross-feature fusion and time series modeling, which enhances detection reliability while reducing both missed detections and false alarms.

#### 3.2.4. Metrics of ROC Curve

Receiver operating characteristic (ROC) curves provide a graphical representation of classification model performance under different decision thresholds. In the context of fire detection, the ROC curve illustrates the relationship between the true positive rate (TPR) and the false positive rate (FPR). Additionally, the area under the ROC curve, known as the area under the curve (AUC), quantifies the discriminative ability of the detection algorithm. The AUC value typically falls between 0.5 and 1, with higher values indicating better classification performance.

**Figure 6:**
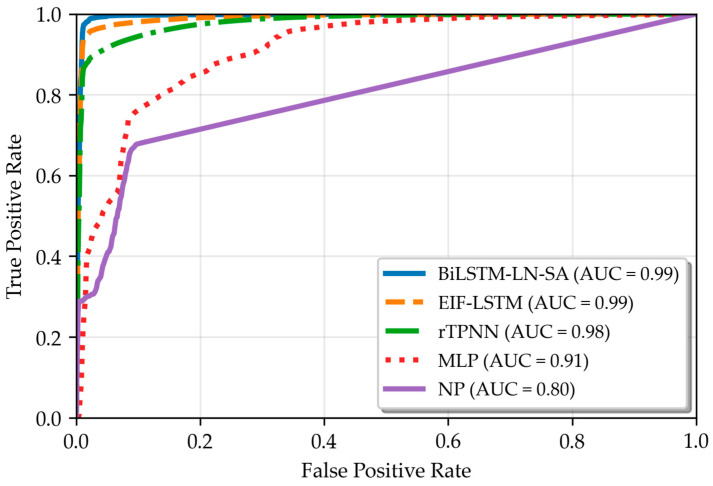
Comparison of different multi-sensor methods based on AUC values.

As shown in Figure 6, BiLSTM-LN-SA achieved an AUC of 0.99, with its ROC curve approaching the top-left corner, which indicates good discriminative capability in distinguishing between fire and non-fire events. The rTPNN model followed with an AUC of 0.98, still maintaining respectable performance though its ROC curve lies slightly below that of BiLSTM-LN-SA and EIF-LSTM. In contrast, MLP (Multilayer Perceptron) and NP attained AUC values of only 0.91 and 0.80, respectively. These lower values indicate their more limited representational capacity in complex fire scenarios and their consequent poorer overall discrimination.

#### 3.2.5. Analysis of the Relationship Between Step Size and Performance in Fire Detection Systems

**Figure 7:**
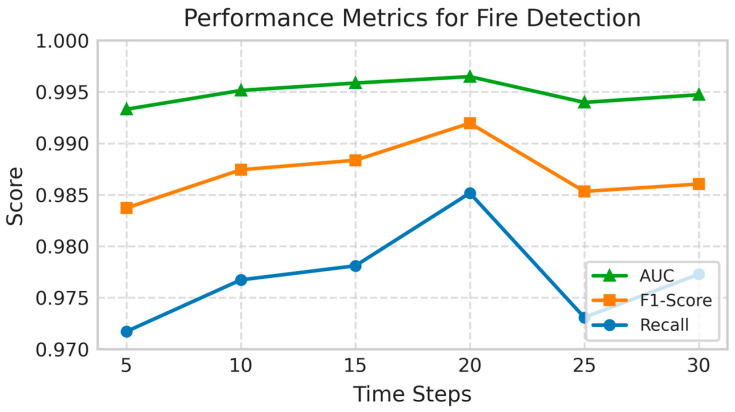
Impact of time step size (in increments of 5) on fire detection performance metrics, including AUC, F1-score, and Recall.

This section investigates the influence of time-series resolution on model performance through three key metrics: AUC, F1-score, and Recall. As shown in Figure 7, a systematic evaluation was conducted at 5-step intervals across a range of 5 to 30 time steps to identify the optimal granularity for fire detection.

All three metrics exhibit consistent optimization trends, reaching peak performance at 20 time steps. The AUC exhibits a non-linear improvement, rising from 0.993 at 5 time steps to a maximum of 0.996 at 20 time steps, before slightly declining to 0.994 at 25 time steps. Similarly, the F1-score follows a comparable trajectory, increasing from an initial value of 0.983 at 5 time steps to an optimum of 0.992 at 20 time steps, indicating a balance between precision and recall. Recall also attains its highest value (0.985) at 20 time steps, reflecting a noticeable improvement from the baseline of 0.972 at 5 time steps.

These results empirically establish 20 time steps as the optimal time-series resolution, achieving an effective trade-off between feature preservation and noise reduction. This configuration enables simultaneous optimization of discriminative capability (AUC), classification robustness (F1-score), and detection sensitivity (Recall), representing a noticeable improvement over both finer and coarser time-series resolutions.

#### 3.2.6. Ablation Study: The Impact of Layer Normalization

To empirically evaluate the effectiveness of the Layer Normalization (LN) module in mitigating feature distribution shifts in multi-scenario fire detection, an ablation study was designed. By comparing the performance of the complete model (BiLSTM-LN-SA) against a variant with the LN layer removed (BiLSTM-SA), this study aims to clarify the contribution of LN to the model’s generalization capability and stability. The two models share identical structures except for the LN module and were trained under the same data and experimental conditions to ensure a fair comparison.

The experiment focused on the most critical performance metrics for fire detection: the false positive rate (FPR) and false negative rate (FNR). As shown in Table 5, all performance metrics were improved with the incorporation of layer normalization. The complete model (BiLSTM-LN-SA) achieved an FPR of 1.50%, which is substantially lower than the 4.80% observed in the model without LN. Meanwhile, the FNR decreased from 3.20% to 1.85%. In addition, the true positive rate (TPR) and true negative rate (TNR) increased to 98.15% and 98.50%, respectively, indicating enhanced discriminative ability for both positive and negative samples.

These results demonstrate that layer normalization effectively mitigates covariate shift caused by varying environmental conditions by stabilizing feature distributions in deep networks. This reduces the model’s sensitivity to absolute feature values and enables it to focus more on discriminative relative patterns and internal feature relationships. Consequently, this mechanism improves the model’s generalization capability and robustness.

#### 3.2.7. Ablation Study: The Impact of Self-Attention Mechanism

To systematically evaluate the effectiveness of the Self-Attention (SA) mechanism in enhancing time-series feature learning for multi-scenario fire detection, an ablation study was conducted. By comparing the performance of the complete model (BiLSTM-LN-SA) against a variant with the self-attention module removed (BiLSTM-LN), this study aims to clarify the specific contribution of the self-attention mechanism to the model’s representational capacity and detection performance. Both models were trained under identical data and experimental conditions, differing only in the inclusion of the self-attention module, ensuring a fair and focused comparison.

The evaluation focused on the most critical performance metrics for fire detection. As shown in Table 6, the integration of the self-attention mechanism led to substantial improvements. The complete model (BiLSTM-LN-SA) achieved an FPR of 1.50%, which is substantially lower than the 5.10% observed in the model without self-attention, representing a 70% reduction in false alarms. Concurrently, the FNR decreased from 7.60% to 1.85%, indicating a significantly improved ability to detect actual fire events. Furthermore, both the TPR and TNR showed marked enhancement, increasing from 92.40% to 98.15% and from 94.90% to 98.50%, respectively. These consistent improvements underscore the critical role of the self-attention mechanism in enhancing the model’s discriminative capability for both fire and non-fire events.

These results demonstrate that the self-attention mechanism significantly enhances the model’s capacity to dynamically weight and integrate the most discriminative time steps within sensor data sequences. By enabling the model to focus on critical time-series intervals indicative of fire events, the self-attention mechanism refines feature representations and suppresses irrelevant variations. This leads to a more precise and robust detection performance, as evidenced by the simultaneous and substantial reduction in both false alarms and missed detections. Consequently, the self-attention mechanism proves to be an essential component for achieving high performance and operational reliability in complex, multi-scenario fire detection tasks.

#### 3.2.8. Comprehensive Discussion: The Complementary Roles of Layer Normalization and Self-Attention

The preceding ablation studies individually evaluated the contributions of the Layer Normalization (LN) and Self-Attention (SA) mechanisms within the BiLSTM-LN-SA model. This subsection provides a comparative analysis of both results to elucidate the distinct roles and synergistic effects of these two components.

As evidenced by Table 5 and Table 6, the removal of either component leads to performance degradation, yet the patterns of decline are markedly different, revealing their fundamental functional distinctions:

(1) Self-Attention as the Core Feature Extractor: Removing the SA mechanism resulted in severe and comprehensive performance deterioration, particularly in critical safety metrics. The False Positive Rate (FPR) increased from 1.50% to 5.10%, while the False Negative Rate (FNR) experienced a substantial rise from 1.85% to 7.60%. This underscores SA’s role as the primary driving component for achieving high model accuracy. By dynamically focusing on fire-critical time steps, it empowers the model to extract highly discriminative features from complex time-series data. The absence of SA critically impairs the model’s capacity to capture essential fire dynamics.
(2) Layer Normalization as the Stability Enhancer: In contrast, the removal of LN led to a more moderate performance decline, with FPR and FNR increasing to 4.80% and 3.20%, respectively. This pattern indicates that LN’s primary function is not to directly enhance representational power, but to act as a stability and generalization enhancer. It mitigates covariate shift induced by varying environmental conditions by stabilizing feature distributions within the deep network. This ensures more reliable learning and enables the model to focus on relative feature patterns rather than absolute values.
(3) Synergistic Interaction Analysis: Together, the two ablation studies reveal a crucial synergistic relationship: the efficacy of LN is predicated on the high-quality time-series features extracted by SA. SA is responsible for generating discriminative feature representations, upon which LN operates to optimize the training process via internal normalization. This allows components like SA to function more stably and efficiently. This division of labor enables the BiLSTM-LN-SA model to achieve both high sensitivity and strong robustness in complex, multi-scenario environments.

The self-attention mechanism serves as the cornerstone for the model’s superior detection performance, while layer normalization acts as the crucial enabler that ensures this performance is sustained reliably across diverse real-world conditions. Their functions are complementary yet distinct, collectively constituting a powerful and dependable fire detection system.

## 4. Conclusions

To address the persistent challenges of high false alarm and missed detection rates in multi-sensor fire detection within complex environments, this paper proposes a novel BiLSTM-LN-SA model that integrates a Bidirectional Long Short-Term Memory (BiLSTM) network with Layer Normalization (LN) and a self-attention (SA) mechanism. The core innovation lies in its synergistic combination of BiLSTM’s capacity for capturing long-term bidirectional dependencies, LN’s ability to stabilize feature distributions and enhance cross-scenario generalization, and SA’s strength in performing dynamic feature enhancement through time-step weighting. This integration enables comprehensive mining and adaptive fusion of deep time-series features from multi-sensor data, significantly improving the accuracy of fire event recognition.

Extensive experimental results on a real-world multi-scenario fire dataset demonstrate the superior performance of the proposed BiLSTM-LN-SA model. It achieves a test accuracy of 98.38%, an F1-score of 0.98, and an AUC of 0.99, significantly outperforming existing methods including MLP, rTPNN, and EIF-LSTM. Crucially, the model maintains a low false positive rate of 1.50% and a false negative rate of 1.85%, effectively balancing the critical trade-off between false alarms and missed detections. Ablation studies confirm the substantial and complementary contributions of both the LN and SA modules: SA serves as the core mechanism for discriminative time-step weighting, while LN enhances training stability and cross-scenario generalization. Additionally, the hyperparameter analysis identifies 20 time steps as the optimal sequence length, achieving the best trade-off between feature preservation and noise reduction.

This research presents a novel and efficient deep learning-based solution for fire detection. Its outstanding real-time perception performance and enhanced resilience to environmental variations demonstrates significant potential for enhancing the reliability of fire monitoring systems in complex scenarios. Future work will focus on developing lightweight versions of the model for deployment on edge computing devices and investigating its potential for extension towards earlier stage fire warning capabilities.

## Figures and Tables

**Table 1:** Comparison of Detection Accuracy Using Multi-Sensor Data.

Methods	Training		Test	
	Mean	Std	Mean	Std
BiLSTM-LN-SA	98.50	0.31	98.38	0.38
EIF-LSTM	96.15	0.41	95.30	0.49
rTPNN	94.10	2.32	93.85	2.16
MLP	87.95	2.02	88.27	2.41
NP	80.05	1.21	80.12	1.26

Note: Results are presented as percentages (%).

**Table 2:** Comparison of Detection Accuracy Using Individual Sensor Data.

Sensor Type	Training		Test	
	Mean	Std	Mean	Std
Temperature	50.62	2.26	50.52	2.29
TVOC	85.89	0.91	85.75	0.80
Carbon dioxide	79.30	2.32	79.25	2.28
NC2.5	84.95	1.36	84.72	1.30

**Table 3:** Performance Comparison of Multi-Sensor Fusion Methods.

Methods	TPR	FNR	TNR	FPR
BiLSTM-LN-SA	98.15	1.85	98.50	1.50
EIF-LSTM	95.20	4.80	96.90	3.10
rTPNN	91.27	8.73	95.27	4.73
MLP	85.27	14.73	91.27	8.73
NP	75.05	25.95	81.25	18.75

**Table 4:** Performance Comparison of Single-Sensor Methods.

Sensor Type	TPR	FNR	TNR	FPR
Temperature	28.30	71.70	98.55	1.45
TVOC	85.40	14.60	99.10	0.90
Carbon dioxide	52.30	47.70	96.80	3.20
NC2.5	82.90	17.10	98.71	1.29

**Table 5:** Performance Comparison of Models with and without Layer Normalization.

Methods	TPR (%)	FNR (%)	TNR (%)	FPR (%)
BiLSTM-LN-SA	98.15	1.85	98.50	1.50
BiLSTM-SA	95.80	3.20	95.20	4.80

**Table 6:** Performance Comparison of Models with and without The Self-Attention Mechanism.

Methods	TPR (%)	FNR (%)	TNR (%)	FPR (%)
BiLSTM-LN-SA (with SA)	98.15	1.85	98.50	1.50
BiLSTM-LN (without SA)	92.40	7.60	94.90	5.10

## Data Availability

The original contributions presented in the study are included in the Article. Further inquiries can be directed to the corresponding author.
